# The Effectiveness of Mindfulness-Based Stress Reduction on Perceived Pain Intensity and Quality of Life in Patients With Chronic Headache

**DOI:** 10.5539/gjhs.v8n4p142

**Published:** 2015-08-06

**Authors:** Nour-Mohammad Bakhshani, Ahmadreza Amirani, Hamed Amirifard, Mahnaz Shahrakipoor

**Affiliations:** 1 Children and Adolescent Health Research Center, Zahedan University of Medical Sciences, Department of Clinical Psychology, Zahedan, Iran; 2 Student Scientific Research Center, Zahedan University of Medical sciences, Department of Clinical Psychology, Zahedan, Iran; 3 Department of Medicine, Zahedan University of Medical Sciences, Zahedan, Iran; 4 Department of biological statistics, Faculty of Health, Zahedan University of Medical sciences, Zahedan, Iran

**Keywords:** chronic pain, migraine headache, mindfulness, quality of life, tension headache

## Abstract

The aim of this study was to determine the effectiveness of Mindfulness-Based Stress reduction (MBSR) on perceived pain intensity and quality of life in patients with chronic headache. Thus, forty patients based on the diagnosis of a neurologist and diagnostic criteria of the International Headache Society (IHS) for migraine and chronic tension-type headache were selected and randomly assigned to the intervention group and control group, respectively. The participants completed the Pain and quality of life (SF-36) questionnaire. The intervention group enrolled in an eight-week MBSR program that incorporated meditation and daily home practice, per week, session of 90-minutes. Results of covariance analysis with the elimination of the pre-test showed significantly improvement of pain and quality of life in the intervention group compared with the control group. The findings from this study revealed that MBSR can be used non-pharmacological intervention for improvement the quality of life and development of strategies to cope with pain in patients with chronic headache. And can be used in combination with other therapies such as pharmacotherapy.

## 1. Introduction

Headache is one of the most common complaints investigated in adult and pediatric neurological clinics. The vast majority of these headaches are migraine and tension-type headaches (Kurt & Kaplan, 2008). Headaches are classified into two categories of main or primary and secondary headaches. Ninety percent of headaches are primary headaches, among which migraine and tension headaches are the most common types (International Headache Society [IHS], 2013). According to the definition, migraine headache is usually unilateral and pulsating in nature and lasts from 4 to 72 hours. The associated symptoms include nausea, vomiting, increased sensitivity to light, sound and pain, and it generally increases with increasing physical activity. Also, tension headache is characterized by bilateral, non-pulsating pain, pressure or tightness, blunt pain, like a bandage or a hat, and a continuum of mild to moderate pain, preventing daily life activities (IHS, 2013).

Stovner et al. (2007) using the IHS diagnostic criteria, estimated the percentages of the adult population with an active headache disorder about 46% for headache in general, 42% for tension-type headache. This suggests that the incidence and the prevalence of tension-type headache are much higher than it was predicted. It is estimated that about 12 to 18 percent of the people have migraines (Stovner & Andree, 2010). Women are more likely to experience migraines compared to men, migraine prevalence is about 6% for men and 18% for women (Tozer et al., 2006).

Migraine and tension-type headaches are common and well-documented responses to psychological and physiological stressors (Menken, Munsat, & Toole, 2000). Migraine is a periodic and debilitating chronic pain and has a negative impact on quality of life, relationships and productivity. The World Health Organization (WHO) has announced the severe migraine as one of the most debilitating diseases with the nineteenth rank (IHS, 2013; Menken et al., 2000).

Despite the development of many medications for treatment and prevention of migraine attacks, a number of patients find them ineffective and some other find them inappropriate because of their side effects and side-effects often times lead to early discontinuation of treatment. As a result, a great interest in the development of non-pharmacologic treatments can be observed (Mulleners, Haan, Dekker, & Ferrari, 2010).

Biological factors alone cannot explain vulnerability to the experience of the headache, the onset of the attack and its course, intensified attacks of headache, headache-related disability and also the quality of life in patients with chronic headache. Negative life events are (as psychosocial factor) often known as a key factor in the development and exacerbation of headache (Nash & Thebarge, 2006).

The program of Mindfulness-Based Stress reduction (MBSR) is among the treatments, which have been studied in the past two decades on a variety of chronic pain. MBSR developed by Kabat-Zinn and used in a wide range of population with stress-related disorders and chronic pain (Kabat-Zinn, 1990). Especially in recent years, many studies have been conducted to examine the therapeutic effects of MBSR. Most studies have shown the significant effects of MBSR on different psychological conditions including the reduction of psychological symptoms of distress, anxiety, rumination, anxiety and depression (Bohlmeijer, Prenger, Taal, & Cuijpers, 2010; Carlson, Speca, Patel, & Goodey, 2003; Grossman, Niemann, Schmidt, & Walach, 2004; Jain et al., 2007; Kabat-Zinn, 1982; Kabat-Zinn, Lipworth, & Burney, 1985; Kabat-Zinn et al., 1992; Teasdale et al., 2002), pain (Flugel et al., 2010; Kabat-Zinn, 1982; Kabat-Zinn et al., 1985; La Cour & Petersen, 2015; Rosenzweig et al., 2010; Zeidan, Gordon, Merchant, & Goolkasian, 2010) and quality of life (Brown & Ryan, 2003; Carlson et al., 2003; Flugel et al., 2010; Kabat-Zinn, 1982; La Cour & Petersen, 2015; Morgan, Ransford, Morgan, Driban, & Wang, 2013; Rosenzweig et al., 2010).

Bohlmeijer et al. (2010) conducted a meta-analysis of eight randomized controlled studies on the effects of MBSR program, concluded that MBSR has small effects on depression, anxiety and psychological distress in people with chronic medical diseases. Also Grossman et al. (2004) in a meta-analysis of 20 controlled and uncontrolled studies on the effects of the MBSR program on physical and mental health of medical and non-medical samples, found an effect size of moderate for controlled studies on mental health. No effect sizes for specific symptoms such as depression and anxiety were reported. The most recent review includes 16 studies controlled and uncontrolled, This review reports that MBSR intervention decrease pain intensity, and most controlled trial studies (6 of 8) show higher reductions in pain intensity for intervention group compared with control group (Reiner, Tibi, & Lipsitz, 2013).

In another study, researchers found significant effect sizes for some subscales of quality of life for example vitality scale and bodily pain, nonsignificant effect sizes for pain and significant medium to large size effects for lower general anxiety and depression (La Cour & Petersen, 2015). Also in a study by Rosenzweig et al. (2010) on patients with chronic pain including those suffering from migraine, there were significant differences in pain intensity, pain-related functional limitations between patients. However, those suffering from migraine experienced the lowest improvement in pain and different aspects of quality of life. In general, different groups of chronic pain showed significant improvements in pain intensity and pain-related functional limitations in this study. Two other studies were conducted by Kabat-Zinn and using MBSR methods for treating patients with chronic pain, including a number of patients with chronic headaches. Statistical analysis showed a significant reduction in pain, pain interference with daily activities, medical and psychiatric signs and symptoms, anxiety and depression, negative body image, pain interference with daily activities, use of the drug and also increase in confidence (Kabat-Zinn, 1982; Kabat-Zinn et al., 1985).

Due to pain and loss of function and reduced work productivity and increased use of health care, chronic headache impose costs on individual and society, it seems that the chronic headache is a major health problem and finding ways to control and treat this problem could be of great importance. The main objective of this study was to evaluate the effectiveness of MBSR in addition to conventional pharmacotherapy in a clinical population sample of patients with chronic headache to show the effectiveness of this technique as a method of pain management and enhancement of the quality of life in patients with chronic headaches.

## 2. Methods

### 2.1 Participants and Procedure

This is a randomized controlled trial two- group ‘pretest-posttest’ study design. Also an approval was obtained from the Ethics Committee of Zahedan University of Medical Sciences. The participants selected through convenience sampling method from patients with chronic migraine and tension-type headache, diagnosed by a neurologist and a psychiatrist using IHS diagnostic criteria-referred to university hospitals of Zahedan University of Medical Sciences, Zahedan-Iran.

After evaluating each patient for meeting the inclusion and exclusion criteria and taking an initial interview, 40 out of eighty-seven primary patients with chronic headache were selected and randomly assigned into two equal groups of intervention and control. Both the control and intervention groups received common pharmacotherapy under the supervision of the neurologist. During therapy sessions three subjects, due to the lack of a regular presence or exclusion criteria, opted out or were excluded from the study.

### 2.2 Inclusion Criteria

(1) Informed consent to participate in the sessions.
(2) Minimum age of 18 years.
(3) Minimum educational qualification of middle-school degree.
(4) The diagnosis of chronic headache (primary chronic migraine and tension-type headache) by the neurologist and according to IHS diagnostic criteria.
(5) 15 or more days per month for more than 3 months and least six months history of migraines and tension-type headache

### 2.3 Exclusion Criteria

(1) Subjects who were not willing to continue the participation in the study or leave the study for any reason.
(2) Other chronic pain problems.
(3) Psychosis, delirium and cognitive disorders.
(4) Cases of interpersonal difficulties interfering with teamwork.
(5) Drug and substance abuse.
(6) Mood disorder

### 2.4 Intervention Groups

Therapy sessions (MBSR) were held for 1.5 to 2 hours a week for the members of the intervention group (drug plus MBSR); While no MBSR was performed for the control group (only common drugs used) until the end of the research. The MBSR was carried out for 8 weeks. In this study, the 8-session MBSR program (Chaskalon, 2011) has been used. To do the meditation homework while training participants in sessions, the necessary measures have been provided in a CD and a booklet. If any one of subjects did not participate in a session or sessions, at the beginning of the next session the therapist would provide written notes of the sessions to the subjects, in addition to repeat the previous session summaries. MBSR program and discussions were presented to the patients in the eight sessions including: understanding pain and its aetiology, discuss about relationship stress, anger and emotion with pain, Understanding negative automatic thoughts, identyfying thoughts and feelings, introducing the concept of Acceptance, breathing space, three-minute breathing space, breath focus exercise, pleasant and unpleasant events daily, behavioral activation, mindfulness of routine activity, body scan practice, Seeing and hearing exercise, sitting meditation, mindful walking, reading poems related to mindfulness and also discuss how to keep up what has been developed over the whole course, discuss plans and positive reasons for maintaining the practice. Patients also received information about learning how to detect any future relapses as well as strategies and plans on which to base early detection of symptom pain attacks and for being self-directed towards new situations.

### 2.5 Controll Group

Patients who were randomized in the control group were continuing usual pharmacotherapy(including specific and nonspecific drugs) by their neurologist until the end of the research.

### 2.6 Instruments

Two main tools were used in the pre-test and post-test to collect data, in addition to demographic data form. Headache log was used to determine the perceived intensity of pain using three parts: (1) 10-point likert-scale ratings, (2) the number of hours of pain per day and (3) pain frequency during the month. Each part is scored from 0 to 100, the highest level being 100. Since each patient rates their perceived pain intensity in the questionnaire, validity and reliability are not considered. And the other was a short-form 36 questionnaire (SF-36). The questionnaire is applicable in the various age groups and different diseases. The reliability and validity of the questionnaire was approved by Ware et al (Ware, Osinski, Dewey, & Gandek, 2000). The SF-36 assesses the perception of the quality of life in 8 subscales include: physical functioning (PF), role limitations due to physical health (RP), bodily pain (PB), general health (GH), energy and vitality (VT), social functioning (SF), role limitations due to emotional problems (RE) and affect health (AH). The tool has also two summary scales for Physical Component Summary (PCS) and Mental Component Summary (MCS) scores. Each scale is scored from 0 to 100, the highest functional status level being 100. The validity and reliability of the SF-36 were examined in an Iranian population. Internal consistency coefficients were between 0.70 and 0.85 for the 8 subscales and test-retest coefficients were between 0.49 and 0.79 with an interval of one week (Montazeri, Goshtasebi, Vahdaninia, & Gandek, 2005).

### 2.7 Data Analysis

For analyzing the data, in addition to the use of descriptive indicators, to compare the results of the intervention and control groups, the analysis of covariance was used to determine the effectiveness and the removal of the pre-test results at 95% confidence level.

#### 2.7.1 Drop-Out

During therapy sessions three subjects, due to the lack of a regular presence or exclusion criteria, opted out or were excluded from the study. Thirty-seven out of 40 patients completed current study and the gathered data were then analyzed.

## 3. Results

Analysis for comparison of demographic distribution between the two groups was performed using chi-square and independent t-test. Demographic data of both groups are shown in Table 1. Distribution of age, educational years, gender and marital status were the same in each group.

**Table 1:** Demographic characteristics of participants^a^

Demographic data	Intervention group	Control group	*P*-value
Age	30.60(9.08)	31.50(9.57)	0.762
Educational years	12.90(3.07)	11.50(3.83)	0.206
Gender			0.824
Male	6(30)	7(35)	
Female	14(70)	13(65)	
Marital status			0.731
Married	12(60)	13(65)	
Single	7(35)	7(35)	
Divorced	1(5)	0(0)	

a Data are presented as mean (SD) or No. (%).

Table 2 shows the results of analysis of covariance (ANCOVA). Levene’s test was non-significant, *F* (1, 35) = 2.78, *P* = 0.105, indicating that the assumption of homogeneity of variance had been approved. This finding shows that the variances across groups are equal and no difference was observed between two groups.

**Table 2:** The results of covariance analysis for the effectiveness of MBSR on pain intensity

Dependent Variable	Source	Sum of Squares	df	Mean Sqare	F	*p*	Partial Eta Squared	Observed power
	Pretest of Pain	7145.25	1	7145.25	73.41	0.001	0.68	0.99
Pain Intensity	Group	2985.80	1	2985.80	30.68	0.001	0.47	0.99
	Error	3309.13	34	97.32	-	-	-	-

The main effect of MBSR intervention was significant, *F* (1, 34) = 30.68, *P* = 0.001, partial *η^2^* = 0.47, indicating that the pain intensity was lower after MBSR intervention (Mean = 53.89, SD.E = 2.40) than control group (Mean = 71.94, SD.E = 2.20). The covariate (pre-test of pain) was also significant, *F* (1, 34) = 73.41, *P* = 0.001, partial *η^2^* = 0.68, indicating that level of pain intensity before MBSR intervention had a significant effect on level of pain intensity. In other words, there was a positive relationship in the pain scores between pre-test and post-test. Therefore, the first research hypothesis is confirmed and MBSR treatment on perceived intensity was effective in patients with chronic headache and could reduce the intensity of perceived pain in these patients. All significant values are reported at *p*<0.05.

The second hypothesis of this study is the effectiveness of MBSR technique on quality of life in patients with chronic headache. To evaluate the effectiveness of MBSR technique on quality of life in patients with chronic headaches and eliminating the confounding variables and the effect of pre-test, for the analysis of data, multivariate covariance analysis (MANCOVA) of the dimensions of quality of life is used that Table 3 shows the results of analysis in the intervention group.

**Table 3:** The results of covariance analysis for the effectiveness of MBSR on quality of life

Dependent Variable	Sum of Squares	df	Mean square	F	*P*	Partial Eta Squared	Observed Power
Post-test of PF	35.43	1	35.43	1.05	0.314	0.04	0.17
Post-test of RP	3053.93	1	3053.93	5.67	0.025	0.18	0.63
Post-test of BP	1582.69	1	1582.69	12.62	0.002	0.34	0.93
Post-test of GH	1080.56	1	1080.56	9.44	0.005	0.28	0.84
Post-test of PCS	1025.52	1	1025.52	9.80	0.004	0.28	0.85
Post-test of RE	893.63	1	893.63	1.74	0.199	0.06	0.25
Post-test of VT	1404.07	1	1404.07	12.60	0.002	0.34	0.93
Post-test of AH	2192.88	1	2192.88	39.85	0.001	0.61	0.99
Post-test of SF	230.28	1	230.28	2.35	0.138	0.09	0.31
Post-test of MCS	1043.34	1	1043.34	12.49	0.002	0.33	0.92

The Table 3 shows the results of analysis of covariance (MANCOVA). The following information is needed to understand the results presented in Table 3.

The box’s test was non- significant, *F* = 1.08, *P* = 0.320, indicating that the variance–covariance matrices are the same in two groups and therefore the assumption of homogeneity is met. Also *F* (10, 16) = 3.153, *P* = 0.020, Wilks’ Lambda = 0.33, partial *η^2^* = 0.66, indicating was a significant difference between the pre-test of the groups in the dependent variables.

Levene’s test was non-significant in some of dependent variables including [PF: *F* (1, 35) = 3.19, *P* = 0.083; RF: *F* (1, 35) = 1.92, *P* = 0.174; BP: *F* (1, 35) = 0.784, *P* = 0.382; GH: *F* (1, 35) = 0.659, *P* = 0.422; PCS: *F* (1, 35) = 2.371, *P* = 0.133; VT: *F* (1, 35) = 4.52, *P* = 0.141; AH: *F* (1, 35) = 1.03, *P* = 0.318], indicating that the assumption of homogeneity of variance had been approved in subscales of quality of life and Levene’s test was significant in some of dependent variables including [RE: *F* (1, 35) = 4.27, *P* = 0.046; SF: *F* (1, 35) = 4.82, *P* = 0.035; MCS: *F* (1, 35) = 11.69, *P* = 0.002], showing that the assumption of homogeneity of variance had been broken in subscales of quality of life.

The main effect of MBSR intervention was significant for some of dependent variables including [RP: *F* (1, 25) = 5.67, *P* = 0.025, partial *η^2^* = 0.18; BP: *F* (1, 25) = 12.62, *P* = 0.002, partial *η^2^* = 0.34; GH: *F* (1, 25) = 9.44, *P* = 0.005, partial *η^2^* = 0.28; PCS: *F* (1, 25) = 9.80, *P* = 0.004, partial *η^2^* = 0.28; VT: *F* (1, 25) = 12.60, *P* = 0.002, partial *η^2^* = 0.34; AH: *F* (1, 25) = 39.85, *P* = 0.001, partial *η^2^* = 0.61; MCS: *F* (1, 25) = 12.49, *P* = 0.002, partial *η^2^* = 0.33], these results indicating that subscales of RP, BP, GH, PCS, VT, AH, and MCS were higher after MBSR intervention [RP: Mean = 61.62, SD.E = 6.18; BP: Mean = 48.97, SD.E = 2.98; GH: Mean = 48.77, SD.E = 2.85; PCS: Mean = 58.52, SD.E = 2.72; VT: Mean = 44.99, SD.E = 2.81; AH: Mean = 52.60, SD.E = 1.97; MCS: Mean = 44.82, SD.E = 2.43] than control group [RP: Mean = 40.24, SD.E = 5.62; BP: Mean = 33.58, SD.E = 2.71; GH: Mean = 36.05, SD.E = 2.59; PCS: Mean = 46.13, SD.E = 2.48; VT: Mean = 30.50, SD.E = 2.56; AH: Mean = 34.49, SD.E = 1.80; MCS: Mean = 32.32, SD.E = 2.21].

Nonetheless, the main effect of MBSR intervention was non-significant for some of dependent variables including [PF: *F* (1, 25) = 1.05, *P* = 0.314, partial *η^2^* = 0.04; RE: *F* (1, 25) = 1.74, *P* = 0.199, partial *η^2^* = 0.06; SF: *F* (1, 25) = 2.35, *P* = 0.138, partial *η^2^* = 0.09]. These results indicating, although the means in these subscales of quality of life were higher [PF: Mean = 75.43, SD.E = 1.54; RE: Mean = 29.65, SD.E = 6.02; SF: Mean = 51.96, SD.E = 2.63] than the control group [PF: Mean = 73.43, SD.E = 1.40; RE: Mean = 18.08, SD.E = 5.48; SF: Mean = 46.09, SD.E = 2.40], But Mean difference was non-significant.

In summary, Covariance analysis (MANCOVA) results in Table 3 indicate a statistically significant difference in the scores of subscales of role limitation due to physical health (RP), bodily pain (BP), general health (GH), energy and vitality (VT), Affect health (AH) and sum of physical health dimensions (PCS) and mental health (MCS). And also indicates that there was not a statistically significant difference in subscale scores of physical functioning (PF), role limitations due to emotional problems (RE) and social functioning (SF) in the intervention group. All significant values are reported at *p*<0.05.

## 4. Discussion

This study aimed to evaluate the effectiveness of MBSR on perceived pain intensity and quality of life in patients with chronic headache. The results showed that MBSR treatment was significantly effective on reduction of pain intensity perception. The results of current study are consistent with the results of other researchers who had used the same method for chronic pain (e.g. Flugel et al., 2010; Kabat-Zinn, 1982; Kabat-Zinn et al., 1985; La Cour & Petersen, 2015; Reibel, Greeson, Brainard, & Rosenzweig, 2001; Reiner et al., 2013; Rosenzweig et al., 2010; zeidan et al., 2010). For example, in two studies conducted by Kabat-Zinn, where the MBSR program was used for treating patients with chronic pain by physicians, a number of patients with chronic headache were also included. The first study of the two studies, showed a significant reduction in pain, pain interference with daily activities, medical signs and psychiatric disorders, including anxiety and depression (Kabat-Zinn, 1982). The results of second study showed significant reduction in pain, negative body image, anxiety, depression, pain interference with daily activities, medical symptoms, medication use, and also showed an increase in self-confidence (Kabat-Zinn et al., 1985).

Also, the findings of the current study are consistent with the results of Rosenzweig et al. (2010), their results suggest that MBSR program is effective for reduction, physical pain, quality of life and psychological well-being of patients with various chronic pains and mindfulness is effective on emotional and sensory components of pain perception by self-regulation of attention through meditation activities. Although the results of Rosenzweig et al. (2010) showed that among patients with chronic pain the minimal impact on the reduction in bodily pain and improvement in quality of life was related to patients with fibromyalgia, chronic headache. In another study conducted by Flugel et al. (2010), although positive changes were observed in the frequency and the intensity of pain, the pain reduction was not statistically significant.

In another study, pain severity significantly reduced after the intervention in patients with tension headache. In addition, the MBSR group showed higher scores in mindful awareness in comparison with the control group (Omidi & Zargar, 2014). In a pilot study by Wells et al. (2014), their results showed that MBSR with pharmacological treatment was possible for patients with migraines. Although the small sample size of this pilot study did not provide power to detect a significant difference in the pain severity and migraine frequency, results demonstrated this intervention had a beneficial effect on headache duration, disability, self-efficacy.

In explaining the results of the effectiveness of mindfulness based therapies for pain it can be said, psychological models of chronic pain such as fear-avoidance model showed that the ways by which people interpret their feelings of pain and respond to them are important determinants in the experience of pain (Schutze, Rees, Preece, & Schutze, 2010). Pain catastrophizing is significantly associated with fear and anxiety caused by pain, the cognitive paths through which the fear of pain can be caused and also the pain-related disability is associated and also because the negative cognitive assessment of pain explains 7 to 31% of the variance of the pain intensity. Therefore, any mechanism that can reduce pain catastrophizing or make changes in its process can reduce the perception of pain intensity and the disability caused by that. Schutz et al. (2010) argue that the little mindfulness is the primer of pain catastrophizing. In fact, it seems that the tendency of the individual to engage in the automatic processing processes rather than knowledge-based processes with attention of insufficient flexibility, and lack of awareness of the present moment (Kabat-Zinn, 1990), will cause people to think more about the pain and thus overestimate the resulting risk of it. Thus, little mindfulness allows for the development of negative cognitive evaluation of the pain (Kabat-Zinn, 1990).

Another possible reason may be that the pain acceptance and readiness for change increase positive emotions, leading to a reduction in pain intensity through effects on the endocrine system and the production of endogenous opioids and reduction in pain-related disability or preparing individuals for the use of effective strategies to deal with pain (Kratz, Davis, & Zautra, 2007). Another possible reason to explain the results of the present study in its effectiveness on pain reduction can be the fact that chronic pain is developed due to an overactive stress response system (Chrousos & Gold, 1992). The result is the disturbing of the physical and mental processes. Mindfulness can allow for the access to the frontal cortex and improve it, brain areas that integrate physical and mental functions (Shapiro et al., 1995). The result is the creation of a little stimulation that reduces the intensity and the experience of physical and mental pain. Thus, pain impulses are experienced as feeling of the real pain rather than a negative recognition. The result is the closing of the pain channels that can reduce pain (Astin, 2004).

Mindfulness meditation Reduces Pain Through several Brain Mechanisms and various pathways such as changing of attention in meditation practices might impress both sensory and affective components of pain perception. On the other hand, mindfulness reduces the reactivity to distressing thoughts and feelings that accompany pain perception and strengthen the pain. Also, mindfulness reduces psychological symptoms such as comorbid anxiety and depression and increases parasympathetic activity, which can promote deep muscle relaxation that may reduce pain. Finally, mindfulness may decrease stress and mood dysfunction-related psychophysiologic activation by strengthening reframing negative situation and self-regulation skills. Higher level of mindfulness predicted lower levels of anxiety, depression, catastrophic thinking and disability. Other research has showed that mindfulness has an important role in cognitive and emotional control, and may be useful in reframing negative situations (Zeidan et al., 2011; Zeidan, Grant, Brown, McHaffie, & Coghill, 2012).

The second aim of this study was to determine the effectiveness of the MBSR program on quality of life in patients with chronic headache. This study showed that this treatment was significantly effective on quality of life dimensions, including role limitations due to health status, bodily pain, general health, energy and vitality, emotional health and overall physical and mental health scales. However, the MBSR program could not significantly increase the quality of life in physical functioning, role limitations due to emotional problems and social functioning. It seems apparent from previous and current studies and as well as from the present study that MBSR no effect on physical and social functions. This is likely because that the effects on pain levels in patients with headache are small, and that change is slow. On the other hand, patients with chronic pain have often learned to ignore pain in order to function normally (La Cour & Petersen, 2015). Although, the changes have been in the desired direction and increased the mean scores of the intervention group compared with the control group. These findings are consistent with previous findings (Brown & Ryan, 2003; Carlson et al., 2003; Flugel et al., 2010; Kabat-Zinn, 1982; La Cour & Petersen, 2015; Morgan et al., 2013; Reibel et al., 2001; Rosenzweig et al., 2010).

With regard to the content of the MBSR sessions, this program emphasizes the application of techniques to reduce stress, deal with pain and the awareness of the situation. Giving up the fight and accepting the present situation, without judgment, is the main concept of the program (Flugel et al., 2010). In fact, changes in acceptance without judgment are associated with improvement in quality of life (Rosenzweig et al., 2010). MBSR is aimed to increase awareness of the present moment. The treatment plan is a new and personal way to deal with stress to the individual. External stressors are part of life and cannot be changed, but coping skills and how to respond to the stress can be changed (Flugel et al., 2010). McCracken and velleman (2010) showed that cognitive flexibility and higher mindfulness is associated with less suffering and disability in patients. Patients with chronic pain with higher levels of mindfulness reported less depression, stress, anxiety and pain and also improvement in the self-efficacy and quality of life. Morgan et al. (2013) studying arthritis patients achieved similar results, so that patients with higher levels of mindfulness reported lower stress, depression and higher self-efficacy and quality of life. As noted above it was expected that pain reduction in patients leads to reduced fear and anxiety associated with pain and thereby reduces the resulting functioning limitations. Also, the results of the several studies (Cho, Heiby, McCracken, Lee, & Moon, 2010; McCracken, Gauntlett-Gilbert, & Vowles, 2007; Rosenzweig et al., 2010; Schutz et al., 2010) confirm this finding.

Several studies have been done to evaluate the effectiveness of different types of mindfulness-based treatments on chronic pain, including patients with headache. Unlike other research that examined heterogeneous sets of patients with chronic pain, the advantage of this study is that, it has been only performed on patients with chronic headache.

In the end, it should be acknowledged that there are some limitations in this study such as small sample size, lack of a long-term follow-up program, participants’ medication use and arbitrary treatments; and despite the efforts of researchers, the lack of fully similar pharmacotherapy for all participants can confound the test results and make it difficult to generalize the results. Since the present study is the first of its type in patients with chronic headache in Iran, it is suggested that similar studies should be carry out in this field, with larger sample sizes as possible. And further studies investigate the stability of the treatment results in long-term follow-up periods of time.

## 5. Conclusion

According to the findings of this study it can be concluded that MBSR methods generally are effective on perceived pain intensity and quality of life of patients with chronic headache. Although there was no statistically significant difference in some aspects of quality of life, such as physical functioning, role limitations due to emotional problems and social functioning, but overall changes in mean were desired to the study. Thus the integrating of MBSR treatment with conventional medical therapy in the treatment protocol for patients with chronic headache can be advised. The researcher also believes that despite the shortcomings and deficiencies of current research, this study could be a new approach to the treatment of chronic headache and could provide a new horizon in this field of treatment.
